# A Plane Extraction Approach in Inverse Depth Images Based on Region-Growing

**DOI:** 10.3390/s21041141

**Published:** 2021-02-06

**Authors:** Xiaoning Han, Xiaohui Wang, Yuquan Leng, Weijia Zhou

**Affiliations:** 1 State Key Laboratory of Robotics, Shenyang Institute of Automation, Chinese Academy of Sciences, Shenyang 110016, China; hanxiaoning@sia.cn (X.H.); wangxiaohui1@sia.cn (X.W.); zwj@sia.cn (W.Z.); 2 Institutes for Robotics and Intelligent Manufacturing, Chinese Academy of Sciences, Shenyang 110016, China; 3 University of Chinese Academy of Sciences, Beijing 100049, China; 4 Shenzhen Key Laboratory of Biomimetic Robotics and Intelligent Systems, Department of Mechanical and Energy Engineering, Southern University of Science and Technology, Shenzhen 518055, China; 5 Guangdong Provincial Key Laboratory of Human-Augmentation and Rehabilitation Robotics in Universities, Southern University of Science and Technology, Shenzhen 518055, China

**Keywords:** plane extraction, region growing, RGBD camera, normal estimation

## Abstract

Planar surfaces are prevalent components of man-made indoor scenes, and plane extraction plays a vital role in practical applications of computer vision and robotics, such as scene understanding, and mobile manipulation. Nowadays, most plane extraction methods are based on reconstruction of the scene. In this paper, plane representation is formulated in inverse-depth images. Based on this representation, we explored the potential to extract planes in images directly. A fast plane extraction approach, which employs the region growing algorithm in inverse-depth images, is presented. This approach consists of two main components: seeding, and region growing. In the seeding component, seeds are carefully selected locally in grid cells to improve exploration efficiency. After seeding, each seed begins to grow into a continuous plane in succession. Both greedy policy and a normal coherence check are employed to find boundaries accurately. During growth, neighbor coplanar planes are checked and merged to overcome the over-segmentation problem. Through experiments on public datasets and generated saw-tooth images, the proposed approach achieves 80.2% CDR (Correct Detection Rate) on the ABW SegComp Dataset, which has proven that it has comparable performance with the state-of-the-art. The proposed approach runs at 5 Hz on typical 680 × 480 images, which has shown its potential in real-time practical applications in computer vision and robotics with further improvement.

## 1. Introduction

Planar surfaces are prevalent components of man-made indoor scenes, such as walls, ceilings, and floors. Extracting those planes will help intelligent agents to understand their surroundings and provide a compact way to model them. Thus, plane extraction plays a vital role in practical applications of computer vision and robotics, such as visual odometry [1], SLAM (Simultaneous Localization and Mapping) [2,3], scene segmentation [4], scene understanding [5], and object recognition [6].

In earlier years, it was intuitive to extract planes based on visual cues, with the assumption that pixels in one plane have a similar texture and appearance [7]. However, those methods will fail as planes can have a similar appearance to other parts. In recent years, due to the rapid development of structured light and time-of-flight techniques, consumer RGBD cameras have become popular in practical applications of computer vision and robotics. RGBD cameras can obtain spatial information, and preserve it in an organized manner. With access to spatial information, the 3D structure of an environment can be retrieved based on SfM (Structure from Motion) or SLAM (Simultaneous Localization and Mapping) technology. Thus, many methods have been proposed based on reconstructed point clouds [8,9]. As 3D construction is a time-consuming process, these plane extraction methods run in an off-line regime. In this work, a plane representation is formulated in inverse-depth images, in which each pixel value is the mathematical inverse of the corresponding one in depth images, and based on this, region growing is directly applied on images, avoiding 3D reconstruction of the scene.

Region growing-based approaches are one of the most popular methods of plane extraction, which can take advantage of connection information preserved in the image during exploiting boundaries. In region-growing approaches, the plane model is initialized with the first seed, thus the selection of seeds is crucial in the region-growing algorithm. The DPD (Depth-driven Plane Detection) approach [10] selects seed patches densely throughout the whole image, with higher planarity first [11]. RANSAC-based methods [8,9] selects seeds randomly from the whole sample set. To make a trade-off between coverage and run-time, the grid local seeding strategy is employed in this work, which is a trade-off between coverage and run-time.

Another important factor of region growing is the termination criteria. Pixel-wise region growing includes inliers and refuse outliers around the seed, according to the termination criteria; therefore, the accuracy of region-growing-based approaches depends on termination criteria. Normal coherence usually serves as the termination criterion [1,12], but it is either sensitive to noise or suffers from limited accuracy. DPD [10] avoids this by applying a dynamic point-plane distance with a two-stage refinement. The greedy policy was proposed as an alternative of normal coherence by Feng et al. [13], which assigns a pixel near boundaries to its closest plane. In this paper, a combination of greedy policy and normal coherence achieves a better performance. Thus, both greedy policy and normal coherence are employed, with a reasonably large point-plane distance, in the proposed approach, as they are more accurate and robust to sensor noise.

Briefly, the main contributions of this paper can be summarized as follows:

- Based on the pin-hole model of a camera, a plane representation in an inverse-depth image is formulated, which can save computational cost by avoiding 3D construction of the environment.
- The region-growing-based approach is improved in two ways. Taking two basic factors, namely locality and coverage into consideration. a grid local seeding strategy is applied in the proposed approach to improve exploration efficiency. Moreover, a combination of greedy policy and normal coherence enable it to be robust to noise.
- The accuracy and efficiency of the proposed method is validated through experiments on public datasets, and generated saw-tooth images. In addition. the complexity is analyzed.

The rest of this paper is organized as follows. Related work is introduced in Section 2. The representation of a plane in inverse-depth image is formulated in Section 3, as well the estimation of normals. The implementation of the proposed planed extraction method is detailed in Section 4. In Section 5.1, the proposed method is evaluated for two datasets and real-world scenarios.

## 2. Related Work

Plane extraction has attracted the interest of scholars in the last ten years, and many methods have been proposed. These traditional methods can be categorized into four groups, i.e., cluster-based, RANSAC-based, Hough Transform-based, and region-growing based approaches, according to their process.

RANSAC is a popular iterative model-fitting approach to estimate model parameters from a noisy sample set [14]. In general, seeds are selected at random from the whole sample set to initialize a model, model fitting is carried out recursively until there has been enough iterations. RANSAC is directly implemented in plane extraction in CC-RANSAC [8], while it performs poorly in complex structures, such as stairs. NCC-RANSAC [9] improves CC-RANSAC by performing a normal coherence check.

Cluster-based methods over-segment an organized sample set into plenty of small patches and build a graph to preserve their connection relations. Then, similar neighboring patches are merged as one, based on visual and geometric cues. An agglomerative hierarchical clustering algorithm was presented by Feng et al. [13], which was conducted on a graph initialized with segmentation of an image into regular cells. Marriott et al. [15] employed the K-means algorithm to generate over-segmented patches, and then a piece-wise-linear Gaussian mixture regression model, combined with outlier-trimming and Expectation Maximization, was employed to fit planar models. In the approach proposed by Xing et al. [16], planar-regions are generated by measuring angularity with smoothness and flatness in a top-down manner, which is accelerated by an auto-balanced search tree.

Hough Transform is well known as being capable of detecting particular shapes in images, such as circles and lines, in 2D images by voting in parameter space [17]. It is transferred to three dimensions, by adapting parameter space in a polar coordination system for plane extraction [11].

Region-growing-based approaches are one of the most popular plane extraction approaches. At first, seeds are selected in the sample set to initialize a plane region, and then the region grows by checking their surrounding pixels according to certain consistency criteria. DPD [18] selects seeds by ranking all patches throughout the image. In addition, a dynamic growing threshold function is implemented, to make the proposed method robust to sensor noise. While DPD is a time-consuming algorithm, as DPD generates seeds in a dense way and employs a two-stage refinement mechanism. In the proposed approach, a grid local seeding strategy is applied to improve exploration efficiency, and both greedy policy and normal coherence serve as termination criteria to enable it to find boundaries accurately and robust to noise.

Nowadays, with the development of deep-learning, some rsearch has explored the possibility to extract planes based on deep neural networks. In the approach, proposed by Liu et al. [5], Mask-RCNN is employed to detect planes from a single RGB image. Deep learning-based methods detect planes in a supervised manner and requires massive labeled samples for training.

## 3. Preparation

Before detailing the proposed approach, some preparation work is introduced in this section. When projected into an inverse-depth image through the pin-hole model, the presentation of a plane is formulated in Section 3.1. Next, a local surface normal estimation method in an inverse-depth image is presented.

### 3.1. Plane in Inverse Depth Images

**Figure 1:**
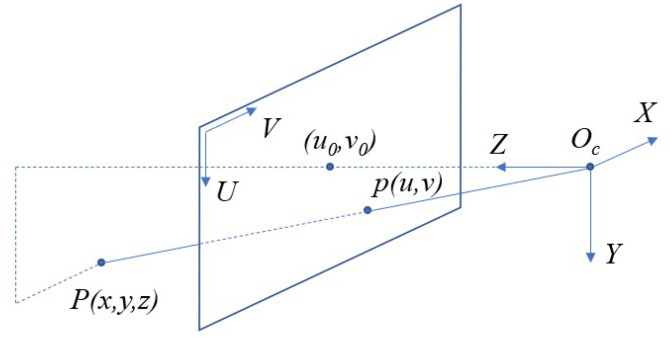
The pin-hole model of a depth camera.

For a depth camera, its imaging process can be described by the pin-hole model, as depicted in Figure 1. A 3D point in the real world is projected onto an image plane through a tiny hole. The model can be expressed as follows:

(1) $$\left\{\begin{array}{cc} u= & \frac{yf_{u}}{z}+u_{0}\\ v= & \frac{xf_{v}}{z}+v_{0}\\ d= & s\cdot z \end{array}\right.$$

where $\left(x,y,z\right)$ is the position of the 3D point in camera coordinate system $O_{c}$, and $\left(u,v\right)$ is the projected pixel in the image with depth value *d*, which is a measurement of depth in *Z* axis, and scaled by a factor *s*. $f_{u}$ and $f_{v}$ are focal length along *U* and *V* axis, respectively. $u_{0}$, and $v_{0}$ are the optical center in the image. The interior parameters of the camera, $\left(k,f_{u},f_{v},u_{0},z_{0}\right)$, can be obtained by a calibration process beforehand. Furthermore, we utilize ρ=1/d to apply Equation (1) in an inverse-depth image.

In, a 3D point can be retrieved by the following:

(2) $$\left\{\begin{array}{cc} x= & \frac{v-v_{0}}{f_{v}s\rho }\\ y= & \frac{u-u_{0}}{f_{u}s\rho }\\ z= & \frac{1}{s\rho } \end{array}\right.$$

In the Cartesian coordinate system, an arbitrary plane can be expressed with a normal vector $n=[n_{A},n_{B},n_{C}]$ and its distance *D* from the original point:

(3) $$n\cdot \left[\begin{array}{c} x\\ y\\ z \end{array}\right]-D=0$$

While in an inverse-depth image, the plane can be formulated with $\left(u,v,\rho \right)$ by substituting Equation (2) into Equation (3):

(4) $$A^{\prime }v+B^{\prime }u+C^{\prime }+D^{\prime }\rho =0$$

where

(5) $$\left\{\begin{array}{cc} A^{\prime }= & \frac{n_{A}}{f_{v}s}\\ B^{\prime }= & \frac{n_{B}}{f_{u}s}\\ C^{\prime }= & \left(\frac{n_{C}}{s}-\frac{n_{A}v_{0}}{f_{v}s}-\frac{n_{B}u_{0}}{f_{u}s}\right)\\ D^{\prime }= & -D \end{array}\right.$$

Compared to the original representation, planes in an inverse-depth image can also be determined by four coefficients. Based on this formulation, we try to extract planes in inverse-depth images, which will be detailed in Section 4.

### 3.2. Estimation of Surface Normals

For plane extraction, surface normal information is a vital geometric cue. The normal of each regular cell is estimated by principal component analysis (PCA) [1]. In this subsection, the local surface normal is calculated from an inverse depth image directly.

Each pixel in an image is a 3D point in the real world, with connectivity and spatial information conserved, the surface normal can be estimated from an inverse-depth image. For a pixel $\left(u,v\right)$ in an image, its surface normal v can be estimated by $\left(\partial z/\partial x,\partial z/\partial y,-1\right)$. According to the chain rule, the former two elements can be deviated by

(6) $$\left\{\begin{array}{cc} \frac{\partial z}{\partial x}= & \frac{\partial z}{\partial u}\cdot \frac{\partial u}{\partial x}+\frac{\partial z}{\partial v}\cdot \frac{\partial v}{\partial x}\\ \frac{\partial z}{\partial y}= & \frac{\partial z}{\partial u}\cdot \frac{\partial u}{\partial y}+\frac{\partial z}{\partial v}\cdot \frac{\partial v}{\partial y} \end{array}\right.$$

According to Equation (2), ∂z/∂u, ∂z/∂v, ∂u/∂x, and ∂v/∂x can be obtained by

(7) $$\left\{\begin{array}{cc} \frac{\partial z}{\partial u} & =-\frac{1}{s\rho^{2}}\cdot \frac{\partial \rho }{\partial u}\\ \frac{\partial z}{\partial v} & =-\frac{1}{s\rho^{2}}\cdot \frac{\partial \rho }{\partial v}\\ \frac{\partial u}{\partial x} & =-s\rho \left(u-u_{0}\right)\cdot \frac{\partial z}{\partial x}\\ \frac{\partial v}{\partial x} & =s\rho f_{v}-s\rho \left(v-v_{0}\right)\cdot \frac{\partial z}{\partial x} \end{array}\right.$$

Then, ∂z/∂x can be obtained by substituting Equation (7) into Equation (6):

(8) $$\frac{\partial z}{\partial x}=\frac{f_{v}\cdot \frac{\partial \rho }{\partial v}}{\left(v-v_{0}\right)\frac{\partial \rho }{\partial v}+\left(u-u_{0}\right)\frac{\partial \rho }{\partial u}-\rho }$$

Similarly, ∂z/∂y can also be obtained

(9) $$\frac{\partial z}{\partial y}=\frac{f_{u}\cdot \frac{\partial \rho }{\partial u}}{\left(v-v_{0}\right)\frac{\partial \rho }{\partial v}+\left(u-u_{0}\right)\frac{\partial \rho }{\partial u}-\rho }$$

In an inverse-depth image, ∂ρ/∂u and ∂ρ/∂v are partial differences in row and column directions, respectively, and can be approximated with

(10) $$\left\{\begin{array}{cc} \frac{\partial d}{\partial u} & =\frac{I_{inv}\left(u+1,v\right)-I_{inv}\left(u-1,v\right)}{2}\\ \frac{\partial d}{\partial v} & =\frac{I_{inv}\left(u,v+1\right)-I_{inv}\left(u,v-1\right)}{2} \end{array}\right.$$

where $I_{inv}\left(u,v\right)$ is the value of pixel (u,v) in the inverse-depth image $I_{inv}$.

Thus, the normal vector $v=\left(\partial z/\partial x,\partial z/\partial y,-1\right)$ of each pixel is calculated, and a normalization operation is implemented to obtain unit normal vector:

(11) $$n=\frac{v}{\left|v\right|}$$

## 4. Approach

**Figure 2:**
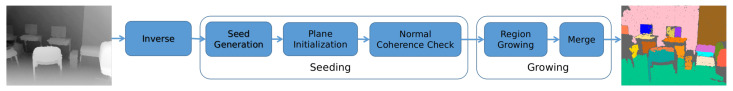
The pipeline of the proposed plane extraction approach, from depth image (**left**) to extraction result (**right**).

In this section, the proposed approach is detailed, as shown in Figure 2. The pipeline consists of two main components: seeding, and region growing.

### 4.1. Grid Local Seeding

The generation and selection of seeds are vital for the initialization of planes and coverage of the image. Instead of select seeds from the whole image randomly, such as the RANSAC-based methods, or generate densely, such as DPD [10]. Seeds are generated and selected by considering the following factors:

- Coverage. In practical applications, without clear intention, a plane may appear anywhere in an image. When trying to extract planes, each plane should be detected and segmented in the whole image.
- Locality. In the real world, planes are usually continuous areas, which do not overlap with each other. When projected onto an image, the adjacent pixels are more likely to be in the same plane.

Based on the factors considered above, a grid local seeding strategy is applied in the proposed approach. To have better coverage, the image is divided into uniform regular cells. Each cell has a size of L×L, thus the image is divided into $\left\lceil \frac{W}{L}\right\rceil \times \left\lceil \frac{H}{L}\right\rceil$ cells. Within each cell, one pixel is selected randomly as a seed. Compared with RANSAC-based methods, seeding in grids can guarantee the whole image is explored evenly.

As three valid pixels are required to initialize a plane, after selecting a seed $s_{0}=\left[u_{0},v_{0},\rho_{0}\right]$ in one grid cell, another two pixels need to be chosen. Considering locality, two pixels, $s_{1}=\left[u_{1},v_{1},\rho_{1}\right]$ and $s_{2}=\left[u_{2},v_{2},\rho_{2}\right]$, are selected in the adjacent local area, which is a circle region around $s_{0}$. On the other hand, to decrease the influence of sensor noise, too close neighboring points are filtered out. In a word, $s_{1}$ and $s_{2}$ are chosen randomly in a ring area around $s_{0}$, by considering locality and sensor noise together.

One plane model can be initialized by the chosen three pixels, which satisfies

(12) $$\left[\begin{array}{c} s_{0}\\ s_{1}\\ s_{2} \end{array}\right]\left[\begin{array}{c} A^{\prime }\\ B^{\prime }\\ C^{\prime }\\ D^{\prime } \end{array}\right]=0$$

With conversion in Equation (5) and unit constraint of *n*, a set of plane coefficients can be determined uniquely, which will be applied in region growing and updated repeatedly.

Although the three pixels are selected from a local adjacent area, there is no guarantee that they are sampled from the same plane. A nonexistent plane would be initialized by three non-coplanar pixels. To avoid such a situation, a normal consistency check is employed after the initialization of a plane to filter out such seeds, which is based on the fact that, in an ideal plane, each point has the same normal vector as the plane. The normal coherence can be measured by:

(13) $$\mu =\min_{i=0,1,2}\left|n_{s_{i}}\cdot n_{p}\right|$$

where $n_{s_{i}}$ is local surface unit normal at pixel $s_{i}$, which is estimated as introduced in Section 3.2, and $n_{p}$ is unit normal vector of the initialized plane.

When the normal coherence of an initialized plane is under a predefined threshold $T_{\mu }$, the seed would be discarded. Otherwise, region growing begins from the seed to its neighbor pixels. To make it robust to sensor noise, $T_{alpha}$ is set to 0.5 empirically.

### 4.2. Plane Growing

**Figure 3:**
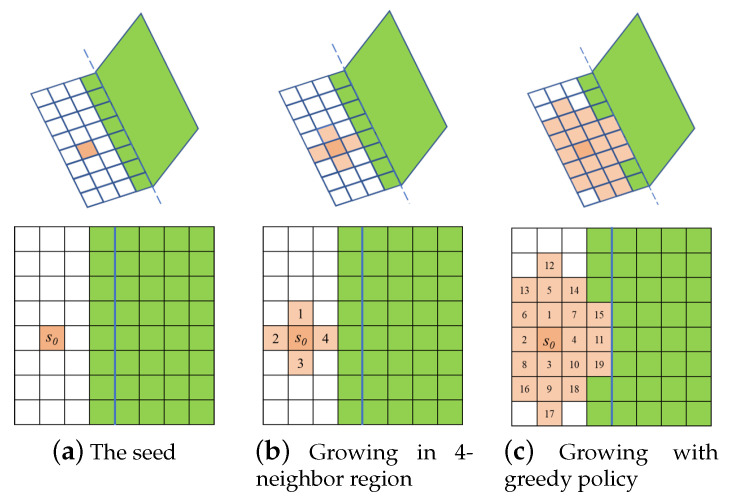
Region growing in four-connected neighbor region with greedy policy.

A pixel-wise region growing algorithm is implemented to extract a continuous plane, once a plane initialized. The region growing begins from the seed, and explores the four connected neighboring points around the region recursively, as depicted in Figure 3. The borders of the region growing are not constrained, thus it can extract the entire plane at once.

The objective of region growing is to include inliers around the seed into the plane until the boundaries are found. In the process of region growing, each potential point is judged as to whether it will be incorporated into the plane by termination cases.

Point-plane distance is the most common termination case for region growing [18]. The point-plane distance can be calculated in an inverse-depth image by

(14) $$\psi =\frac{|A^{\prime }v+B^{\prime }u+C^{\prime }+D^{\prime }\rho |}{\rho \sqrt{1+D^{\prime 2}}}$$

To serve as termination criteria, a distance threshold $T_{\psi }$ is adopted. Other than a fixed threshold value, which leads to either over-segmentation (too small a value) or under-segmentation (too large a value), several dynamic thresholds have been carefully designed as quadratic functions, which increase with distance [10,19]. With dynamic thresholds, the region growing process is robust to sensor noise. The utilization of an increasing dynamic threshold may lead to a problem of over-growing, as shown in Figure 3a. An off-line refinement mechanism is employed to avoid the over-growing problem by calculating the intersection line, and reassigning pixels around it [10]. Differently, in the proposed approach, a greedy policy, with a reasonable large fixed threshold value, is employed in point-plane distance termination case. As shown in Figure 3c, one pixel near the intersection area would be judged several times, as each of the potential planes grows through it, and it will be incorporated into the one with the smallest point-plane distance.

Normal coherence is another popular termination criteria in region growing. Similar to implementation in seed selection, the normal coherence between the explored pixel and the plane is calculated. As suggested by Qian et al. [9], since the normal estimation of one individual pixel is sensitive to sensor noise, the normal coherence threshold for termination criteria is set to 0.37π, which makes it robust to sensor noise and avoids the extension of a mis-initialized plane in the early stage.

The coefficients of a plane are updated to minimize square error in the process of growing, based on PCA. As more points are added to the plane, its coefficients converge gradually. To avoid computing too frequently, the update mechanism runs in a lazy manner, as a log function of the plane scale.

To overcome the over-segmentation problem of large planes, a merging process is conducted to adjoin neighboring potential co-planar planes, once a plane has finished its growing. This process is realized as follows, in the process of plane growing, its neighboring planes are recorded in a candidate set. Each plane is the candidate set is judged as to whether it is a co-planar surface with the grown one, through a co-planar check:

(15) $$\left\{\begin{array}{cc} n_{p_{i}}\cdot n_{p_{j}}<T_{ang}, & \\ |D_{p_{i}}^{\prime }-D_{p_{j}}^{\prime }|<T_{dis}, & p_{i}\in N_{j} \end{array}\right.$$

where $N_{j}$ is the neighboring plane set of plane $p_{j}$, $T_{ang}$ and $T_{dis}$ are thresholds for normal and distance difference, respectively. If two planes are checked as co-planar, they will be merged as one, and the parameters of the novel plane will be refitted and updated with the implementation of PCA.

To avoid generating too many noisy patches, tiny planes are discarded, in which the count of pixels is under a pre-defined scale threshold.

## 5. Experiment

In this section, two public datasets, i.e., SegComp ABW dataset [20], and NYU Depth Dataset V2 [21], and generated saw-tooth images are utilized to evaluate the proposed plane extraction method both qualitatively and quantitatively.

### 5.1. Evaluation on SegComp ABW Dataset

The SegComp ABW Dataset [20] is a well-known dataset for benchmarking different plane extraction methods. This dataset is collected by placing one or a few polyhedral objects onto a horizontal plane, with a vertical plane as the background. This dataset was captured with an ABW camera, which saved images in a different format with popular consumer RGBD cameras, such as Microsoft Kinect and ASUS Xtion. To apply the proposed method in this dataset, we adjust each image in a Kinect-like manner by increasing each pixel by a constant value of 607.55. After such adjustment, the equivalent interior parameters for the SegComp ABW Dataset are listed in Table 1.

The SegComp ABW Dataset consists of two sets, one set contains 10 images for training and the other contains 30 for testing. In this section, the training set was utilized for the ablation study of the proposed approach, and we compared the performance of the proposed approach with other state-of-the-art ones on the testing set.

In this subsection, we conducted two individual ablation studies on both seeding strategies and termination criteria on the training set, to have a prospective look into the proposed method. Then, we compared the proposed methods with some typical and state-of-the-art approaches in the test set.

#### 5.1.1. Seeding Strategies

In this part, we aim to analyze the effects of different seeding strategies. Both greedy policy and normal coherence are the parameters for termination and merge mechanisms and are fixed at $T_{psi}=0.002$, $T_{dis}=0.008$, $T_{ang}=0.1\pi$. As suggested by Gotardo et al. [22], we view one extracted plane, which has more than 80% overlap with a ground truth plane, as a true positive case. To evaluate the exploitation efficiency of different seeding strategies, CDR (Correct Detection Rate) is chosen as an indicator, which is a ratio of true positive detected planes with all labeled ground truth ones.

Four different seeding strategies are compared in this experiment, namely Random, Local, Grid, and Grid Local. The Random strategy is a completely randomized seeding strategy, which is similar to the initialization of RANSAC, and its exploitation efficiency was analyzed by Qian et al. [9] Grid and Local strategy, each has only one individual module of the proposed Grid Local strategy, also serve as benchmarks.

**Figure 4:**
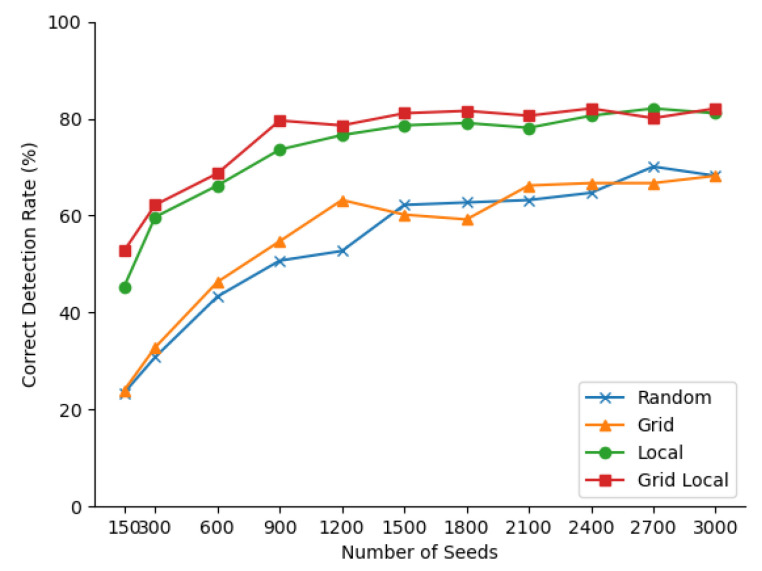
Comparison of different seeding strategies.

The ablation study experiment result of different seeding strategies is shown in Figure 4. It can be found that, for each strategy, as the number of seeds increases from 150 to 1500, the CDR curve gradually increases, which means more planes are detected. Four lines more planes are extracted from images. While for the two strategies with local seeding, CDR curves fluctuate slightly, instead of growing. This is because it reaches its best performance, with some planes challenging to detect, which contain only a few pixels. It can be concluded that the local seeding strategy can improve exploration efficiency significantly. Despite some outliers, the two CDR curves with grid strategy go above the other two, which means that grid strategy also upgrades the performance of the proposed approaches.

#### 5.1.2. Termination Criteria and Merge

When a potential plane grows from a seed, termination criteria determine whether a neighbor pixel should be included as an inlier or not. Thus, the accuracy of the extracted plane depends on the termination conditions. In this part, an ablation study on different combinations of termination criteria and merge mechanism is conducted on SegComp ABW Dataset.

Quantitative analysis is based on the Receiver Operating Characteristic (ROC). As listed in Table 2, *sensitivity*, *specificity*, and CDR, are chosen as. For an extracted plane, ROC indicators can be calculated by sensitivity=#TP/#(TP+FN), and specificity=#TN/#(TN+FP). TP is the count of true positive pixels included in the extracted plane. Similarly, FN, TN, and FP are counts of false-negative, true-positive, and false-positive pixels, respectively.

The experimental results are listed in Table 2. In the process of plane growing, four different combination groups are considered, where three serve as control groups, each of which is absent of individual measures. As depicted in the table, as point-plane distance tolerance increases from 0.001 to 0.003, sensitivity gradually increases, as more pixels with noise are included in planes. Compared with the three control groups, the proposed method with all three measures achieves the best performance, with the highest sensitivity and CDR, which means it detected more planes, also with the highest boundary accuracy.

#### 5.1.3. Comparison with State-of-the-Art

We have compared the proposed approach with several typical ones on SegComp ABW test set with an 80% area overlap tolerance, including USF [22], WSU [22], UB [22], UE [22], OU [22], PPU [22], UA [22], UFPR [22], Trevor et al. [23], Georgiev et al. [24], Holz et al. [25], Oehler et al. [26], Holz et al. [27], and DPD [10]. In this part, 1800 seeds are put in the image with grid local seeding strategy, and the distance threshold is set to 0.025, which achieved the best performance in ablation study on the train set.

**Figure 5:**
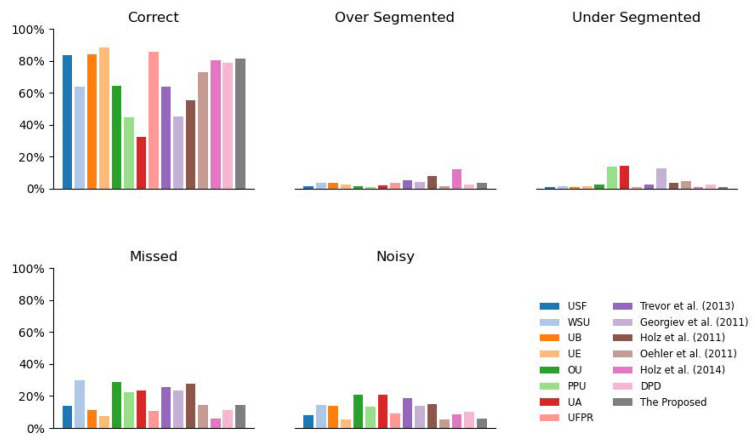
Comparison on test set of SegComp ABW DATASET with 80% overlap tolerance.

In Figure 5, it can be seen that the proposed approach achieves a comparable performance with the state-of-the-art approaches. The proposed approach detects 80.3% of planes correctly, and ranks 5th in all 15 approaches, only falling behind UE (88.1%), UB (84.2%), UFPR (85.5%), and USF (83.5%). The proposed approach also generates a few over-segmented (3.3%, ranking 7/15), under-segmented (0.7%, ranking 1/15), missed (14.5%, ranking 7/15) and noisy (5.9%, ranking 2/15) planes. Though some planes are missed in detected ones, the reason is that there are some planes, which contain only a few pixels and are intractable to handle, even for the dense-seeding approaches. For USF, WSU, and UB, the parameters are obtained by training on the same dataset. Though with several hyper-parameters to determine, the ablation study has shown that the proposed approach has a large optional range. When the count of seeds increases from 1500 to 3000 (shown in Figure 4), and the distance threshold changes from 0.015 to 0.003 (shown in in Table 2), the CDR, sensitivity, and specificity change little. Compared to 7 parameters in UFPR, only 4 parameters need to be determined in the proposed approach, namely seed count, $T_{psi}$, $T_{ang}$, and $T_{dis}$. $T_{psi}$ can be chosen according to sensor noise, which is accessible in most cases, and the others can be selected empirically.

### 5.2. Evaluation on Sawtooth Images

**Figure 6:**
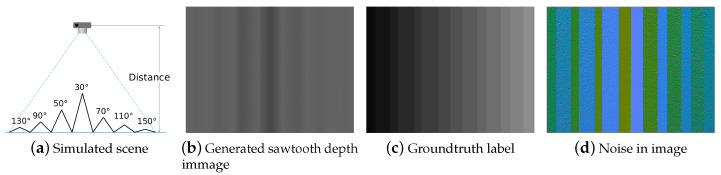
Sawtooth images generated for experiments.

To evaluate the robustness of the proposed approach, a set of sawtooth depth images are generated with a resolution 640×480. As shown in Figure 6a, it is a saw tooth structure with different heights. Three images are generated with distance at 1, 2, and 4 m. To simulate images captured from a consumer RGBD camera, both axial and lateral noise are added into images, according to a specific noise model [28].

Compared with the experimental result on the SegComp ABW dataset, the accuracy performance on sawtooth images is similar, as listed in Table 3. As the point-plane distance threshold increases from 0.001 to 0.003, the sensitivity increases, as it becomes more robust to noisy pixels. While a too large distance threshold may cause the over-growing problem, a normal coherence check and greedy policy can suppress this to a certain extent. Normal estimation is sensitive to noise [10], thus the normal angle threshold is set at a reasonably large value, namely 0.3 to 0.4π.

### 5.3. Evaluation on NYU Depth v2

The images in the NYU Depth v2 dataset were collected with Microsoft Kinect, which captures an RGB and a depth image simultaneously. The images, utilized in this experiment, form a subset of NYU Depth v2, similar to the experiment set up by Jin et al. [10]. Compared with the SegComp ABW dataset and generated sawtooth images, the images in NYU Depth v2 Dataset are captured in real-world scenes, which are more complicated and contain various objects.

#### 5.3.1. Qualitative Comparison

**Figure 7:**
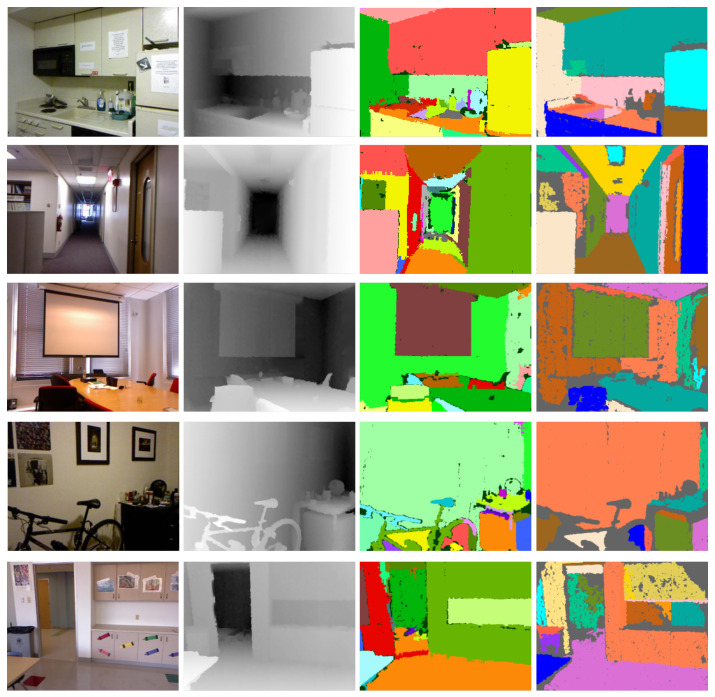
Detection performance comparison of the proposed method and DPD [10]. For each row from (**left**) to (**right**): RGB image, Depth image, the results in DPD and the proposed method.

For the NYU Depth v2 dataset, we evaluated the qualitative performance of the proposed approach with DPD. In Figure 7, a qualitative comparison is shown. The DPD approach is better at detecting large planes. While at the same time, the over-segmentation problem is more common, as the dynamic threshold increases with growing, and some small planes are swallowed. As for the proposed method, as it takes normal information into the growing process, it is sensitive to noise. It would stop growing before the whole plane is segmented, and produce more holes in the planes, as shown in the second row. Another limitation of DPD is that, as it only employed point-plane distance as termination criteria, one earlier extracted plane may truncate other intersecting planes, by including some outliers that belonged to another one. For example, the upper plane of the kitchen counter cuts the front surface of the refrigerator at an inapproachable position. While for the proposed approach, there are some failure cases, as it also tends to over-segment large planes in scenes, such as walls and floors. The reason for this situation is two-fold. Due to radial noise produced in an RGBD camera, large planes are projected curve ones. On the other hand, the lazy update and the constant distance threshold are employed in the proposed approach, which makes it less robust to handle such curve planes.

#### 5.3.2. Computational Complexity Analysis

The computational complexity of the propsed method was analyzed. Taking a depth image, whose size is n=W×H, for example, in the seed selection process, the image is segmented into L×L grid cells, thus the image is divided into ⌈W/L⌉×⌈H/L⌉ cells. For each cell grid, one plane would be initialized. In this way, m=⌈W/L⌉×⌈H/L⌉ planes are initialized as seeds. As the runtime of plane initialization, the computational complexity of the seed selection is O(m). In most cases, the count of seeds is proportional to the image size; therefore, the complexity of the seeding process is O(n). During the region-growing process, the greedy policy and the neighbor search are employed, the complexity is O(nlogn). In a word, the complexity of the whole approach is O(nlogn).

In Table 4, the time cost of each process of the approach is listed, which was run on the NYU depth v2 Dataset. The proposed approach was implemented with C++ on a PC with Intel i5-4590 CPU and 4G RAM. The most time-consuming module is growing, which takes 147.81 ms. The seeding module only takes 4.65 ms for 800 seeds. The total time cost of the approach is 185.68 ms. Compared to NCC-RANSAC and DPD, the time cost of which is about 5 and 20 s, respectively (reported by Jin et al. [10]), the proposed approach has a lower time cost. The approach has the potential for practical applications with faster parallel optimization, as the normal estimation and the region growing can be processed in parallel.

## 6. Conclusions

In this paper, a plane extraction approach based on region growing in inverse-depth images has been proposed and analyzed. Considering the pin-hole model of imaging, a plane representation is formulated in an inverse-depth image. Thus, by growing the inverse-depth image, the reconstruction process is avoided. To improve exploration efficiency, a grid local seeding strategy is employed in the proposed approach. Furthermore, to ensure accuracy and robustness, greedy policy and normal coherence check are combined to include noisy pixels. Then, a merging mechanism serves as a refinement measure to integrate co-planar surfaces into one, to suppress the over-segmentation problem. Through experiments on the SegComp ABW Dataset, generated saw-tooth images, and NYU Depth v2 Dataset, the results show that the proposed method can detect planes in inverse-depth images in an efficient way, with high accuracy and detected rate. The proposed method runs in a time-efficient manner at 5 Hz on typical 640 × 480 images and has the potential to be applied in practical applications.

There are also two limitations in the proposed approach. The first one is that the approach tends to over-segment large planes in scenes, due to radial noise in sensors, and the lazy update of parameters. The second is that it is not fast enough for real-time application. Thus, there are two future directions for the research. A noise model in inverse-depth images would be introduced in the proposed approach, to filter radial noise in an image. On the other hand, the source code will be transferred into a parallel process to speed it up. Furthermore, we will apply the proposed method to object-discovery tasks in structured scenes, in order to build object-oriented semantic maps for robots, combined with object-recognition methods.

## Figures and Tables

**Table 1:** Camera interior parameters for SegComp ABW Dataset.

Interior Parameters	Value
*s*	773.545
$u_{0}$	255
$v_{0}$	255
$f_{u}$	2337.212
$f_{v}$	1610.982

**Table 2:** Performance under different termination conditions.

Distance	G ${}^{1}$	N ${}^{1}$	M ${}^{1}$	Sensitivity	Specificity	CDR
0.001		✓	✓	92.0	99.8	79.1
	✓		✓	94.3	99.9	75.5
	✓	✓		94.3	99.9	74.9
	✓	✓	✓	94.3	99.9	76.3
0.0015		✓	✓	92.5	99.9	80.2
	✓		✓	95.0	99.9	82.7
	✓	✓		95.3	99.9	83.0
	✓	✓	✓	95.3	99.9	83.8
0.002		✓	✓	91.8	99.9	82.8
	✓		✓	95.3	99.9	83.5
	✓	✓		95.7	99.9	84.3
	✓	✓	✓	95.7	99.9	84.9
0.0025		✓	✓	91.3	99.9	79.2
	✓		✓	95.1	99.9	83.9
	✓	✓		95.8	99.9	84.6
	✓	✓	✓	95.9	99.9	85.1
0.003		✓	✓	90.6	99.9	77.4
	✓		✓	94.7	99.9	83.2
	✓	✓		95.8	99.9	83.2
	✓	✓	✓	95.8	99.9	84.4

^1^ G: Greedy Policy, N: Normal Consensus Check, M: Merging.

**Table 3:** Performance on sawtooth images with noise.

Distance	G *	N *	Sensitivity	Speciticity	CDR
0.001			80.9	98.5	54.8
	✓		80.8	98.5	54.8
		✓	83.1	98.7	45.2
	✓	✓	81.8	98.6	47.2
0.002			95.7	99.6	100.0
	✓		96.2	99.7	100.0
		✓	95.9	99.7	100.0
	✓	✓	96.3	99.7	100.0
0.003			96.7	99.7	1.000
	✓		98.0	99.8	100.0
		✓	97.5	99.8	100.0
	✓	✓	98.3	99.9	100.0
0.004			95.1	99.6	100.0
	✓		97.2	99.8	100.0
		✓	97.1	99.7	100.0
	✓	✓	98.3	99.9	100.0

* G: Greedy Policy, N: Normal Consensus Check.

**Table 4:** Average time cost on NYU depth v2 dataset.

Process	Time (ms)
Inverse	1.64
Normal Estimation	34.88
Seeding	4.65
Growing	147.81
Merge	0.06
Total	185.68

## References

[1] Proença P.F., Gao Y. Fast Cylinder and Plane Extraction from Depth Cameras for Visual Odometry. Proceedings of the 2018 IEEE/RSJ International Conference on Intelligent Robots and Systems (IROS).

[2] Yang S., Song Y., Kaess M., Scherer S. Pop-up SLAM: Semantic monocular plane SLAM for low-texture environments. Proceedings of the 2016 IEEE/RSJ International Conference on Intelligent Robots and Systems (IROS).

[3] Yang S., Scherer S. (2019). Monocular Object and Plane SLAM in Structured Environments. IEEE Robot. Autom. Lett..

[4] Pham T.T., Eich M., Reid I., Wyeth G. Geometrically consistent plane extraction for dense indoor 3D maps segmentation. Proceedings of the 2016 IEEE/RSJ International Conference on Intelligent Robots and Systems (IROS).

[5] Liu C., Kim K., Gu J., Furukawa Y., Kautz J. PlaneRCNN: 3D Plane Detection and Reconstruction From a Single Image. Proceedings of the IEEE/CVF Conference on Computer Vision and Pattern Recognition (CVPR).

[6] Deng Z., Todorovic S., Jan Latecki L. (2017). Unsupervised object region proposals for RGB-D indoor scenes. Comput. Vis. Image Underst..

[7] Doulamis A.D., Doulamis N.D., Ntalianis K.S., Kollias S.D. Unsupervised semantic object segmentation of stereoscopic video sequences. Proceedings the 1999 International Conference on Information Intelligence and Systems (Cat. No.PR00446).

[8] Gallo O., Manduchi R., Rafii A. (2011). CC-RANSAC: Fitting planes in the presence of multiple surfaces in range data. Pattern Recognit. Lett..

[9] Qian X., Ye C. (2014). NCC-RANSAC: A Fast Plane Extraction Method for 3-D Range Data Segmentation. IEEE Trans. Cybern..

[10] Jin Z., Tillo T., Zou W., Zhao Y., Li X. (2019). Robust Plane Detection Using Depth Information From a Consumer Depth Camera. IEEE Trans. Circuits Syst. Video Technol..

[11] Tian Y., Song W., Chen L., Sung Y., Kwak J., Sun S. (2020). Fast planar detection system using a GPU-based 3D Hough transform for LiDAR point clouds. Appl. Sci..

[12] Holz D., Behnke S., Lee S., Cho H., Yoon K.J., Lee J. (2013). Fast Range Image Segmentation and Smoothing Using Approximate Surface Reconstruction and Region Growing. Intelligent Autonomous Systems 12.

[13] Feng C., Taguchi Y., Kamat V.R. Fast plane extraction in organized point clouds using agglomerative hierarchical clustering. Proceedings of the 2014 IEEE International Conference on Robotics and Automation (ICRA).

[14] Fischler M.A., Bolles R.C. (1981). Random Sample Consensus: A Paradigm for Model Fitting with Applications to Image Analysis and Automated Cartography. Commun. ACM.

[15] Marriott R.T., Pashevich A., Horaud R. (2018). Plane-extraction from depth-data using a Gaussian mixture regression model. Pattern Recognit. Lett..

[16] Xing Z., Shi Z. (2019). Extracting Multiple Planar Surfaces Effectively and Efficiently Based on 3D Depth Sensors. IEEE Access.

[17] Hough P.V. (1962). Method and Means for Recognizing Complex Patterns. US Patent.

[18] Jin Z., Tillo T., Zou W., Li X., Lim E.G. (2018). Depth image-based plane detection. Big Data Anal..

[19] Holzer S., Rusu R.B., Dixon M., Gedikli S., Navab N. Adaptive neighborhood selection for real-time surface normal estimation from organized point cloud data using integral images. Proceedings of the 2012 IEEE/RSJ International Conference on Intelligent Robots and Systems.

[20] Hoover A., Jean-Baptiste G., Jiang X., Flynn P.J., Bunke H., Goldgof D.B., Bowyer K., Eggert D.W., Fitzgibbon A., Fisher R.B. (1996). An experimental comparison of range image segmentation algorithms. IEEE Trans. Pattern Anal. Mach. Intell..

[21] Silberman N., Hoiem D., Kohli P., Fergus R., Fitzgibbon A., Lazebnik S., Perona P., Sato Y., Schmid C. (2012). Indoor Segmentation and Support Inference from RGBD Images. Computer Vision–ECCV 2012.

[22] Gotardo P.F.U., Bellon O.R.P., Silva L. Range image segmentation by surface extraction using an improved robust estimator. Proceedings of the 2003 IEEE Computer Society Conference on Computer Vision and Pattern Recognition, 2003 Proceedings.

[23] Trevor A.J., Gedikli S., Rusu R.B., Christensen H.I. (2013). Efficient Organized Point Cloud Segmentation with Connected Components.

[24] Georgiev K., Creed R.T., Lakaemper R. Fast plane extraction in 3D range data based on line segments. Proceedings of the 2011 IEEE/RSJ International Conference on Intelligent Robots and Systems.

[25] Holz D., Schnabel R., Droeschel D., Stückler J., Behnke S., Ruiz-del Solar J., Chown E., Plöger P.G. (2011). Towards Semantic Scene Analysis with Time-of-Flight Cameras. RoboCup 2010: Robot Soccer World Cup XIV.

[26] Oehler B., Stueckler J., Welle J., Schulz D., Behnke S., Jeschke S., Liu H., Schilberg D. (2011). Efficient Multi-resolution Plane Segmentation of 3D Point Clouds. Intelligent Robotics and Applications.

[27] Holz D., Behnke S. (2014). Approximate triangulation and region growing for efficient segmentation and smoothing of range images. Robot. Auton. Syst..

[28] Fankhauser P., Bloesch M., Rodriguez D., Kaestner R., Hutter M., Siegwart R. Kinect v2 for mobile robot navigation: Evaluation and modeling. Proceedings of the 2015 International Conference on Advanced Robotics (ICAR).

